# Pneumomediastinum and Pneumothorax as Rare Complications of Ruptured Pulmonary Hydatid Cyst: A Case Report

**DOI:** 10.1002/ccr3.71776

**Published:** 2025-12-30

**Authors:** Farzaneh Akbari, Farid poursadegh, Marzie Noori, Amir Hossein Jafarian, Mahnaz Mozdorian, Fariba Rezaeetalab

**Affiliations:** ^1^ Lung Diseases Research Center Mashhad University of Medical Sciences Mashhad Iran; ^2^ Faculty of Medicine Mashhad University of Medical Sciences Mashhad Iran; ^3^ Cancer and Molecular Research Center, Department of Pathology, Ghaem Hospital, School of Medicine Mashhad University of Medical Sciences Mashhad Iran

**Keywords:** hydatid cyst, Pneumomediastinum, pneumothorax, pulmonary complications

## Abstract

Ruptured hydatid cyst should be considered when encountering spontaneous pneumothorax and pneumomediastinum, especially in young patients in regions with high prevalence of hydatid cyst. Awareness of atypical signs ensures instant diagnosis, guiding appropriate surgical and medical treatment to prevent serious complications.

**Key Clinical Message**

Ruptured pulmonary hydatid cyst can rarely present with simultaneous pneumothorax and pneumomediastinum, even in patients without a typical exposure history especially in an endemic area. Early radiologic evaluation and prompt surgical intervention are essential to prevent life‐threatening complications.

## 1 Introduction

Hydatid disease, a zoonotic parasitic infection caused by *Echinococcus granulosus*, remains endemic in many regions, including the Middle East, Mediterranean, and parts of South America. The liver and lungs are the most commonly involved organs [1]. Pulmonary hydatid cysts are often asymptomatic but may rupture, leading to complications such as pneumothorax, pleural effusion, and rarely pneumomediastinum [2]. Pneumomediastinum, defined as the presence of free air within the mediastinal space, is an uncommon but potentially life‐threatening complication of ruptured hydatid cysts. Awareness and early recognition are essential for optimal management [3].

Herein, we report a rare case of a ruptured pulmonary hydatid cyst complicated by simultaneous pneumothorax and pneumomediastinum.

## 2 Case History/Examination

A 21‐year‐old male presented with a sudden onset of chest pain, progressive shortness of breath, and respiratory distress. The patient denied any history of trauma, smoking, or chronic pulmonary diseases. On examination, vital signs showed tachypnea (30 breaths/min), hypoxia (SpO_2_ 85% on room air), and tachycardia (120 bpm). Physical examination revealed decreased breath sounds on both sides and subcutaneous emphysema over the neck and upper chest.

Lab data show:

WBC = 13.9 x 10^3/μL (4.4–11.3).

RBC = 5.16 x 10^6/μL (4.5–5.9).

Hb = 15.3 g/dL (12.3–15.3).

Platelet = 579 x 10^3/μL (150–450).

Neut = 78.9% (45.5–73.1).

Lymph = 16.0% (20–45).

Mixed (Mono+Eos + Baso) = 5.1% (6–15), HAT for hydatid cyst = 9 (up to 11).

Urea = 24 Cr = 0.9 FBS = 108 mg/dL Blood culture = negative.

The patient was a student and denied any history of contact with dogs or domestic animals, which are familiar sources of echinococcus infection.

## 3 Differential Diagnosis, Investigations and Treatment

**FIGURE 1:**
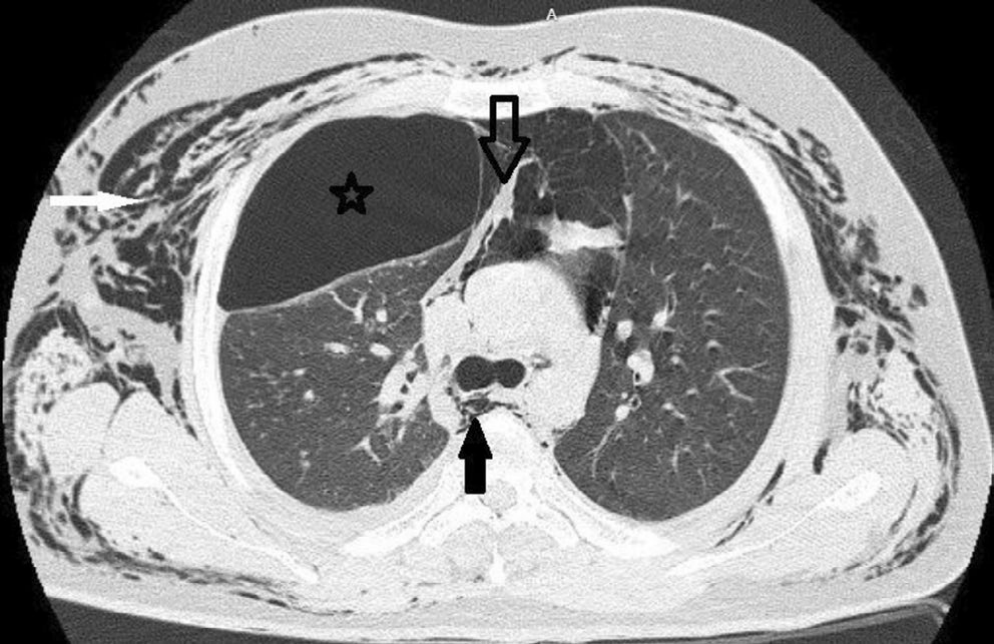
Soft tissue emphysema (white arrow), pneumothorax (asterisk), pneumomediastinum (black arrow), ruptured hydatid cyst (hollow arrow), in lung CT scan.

**FIGURE 2:**
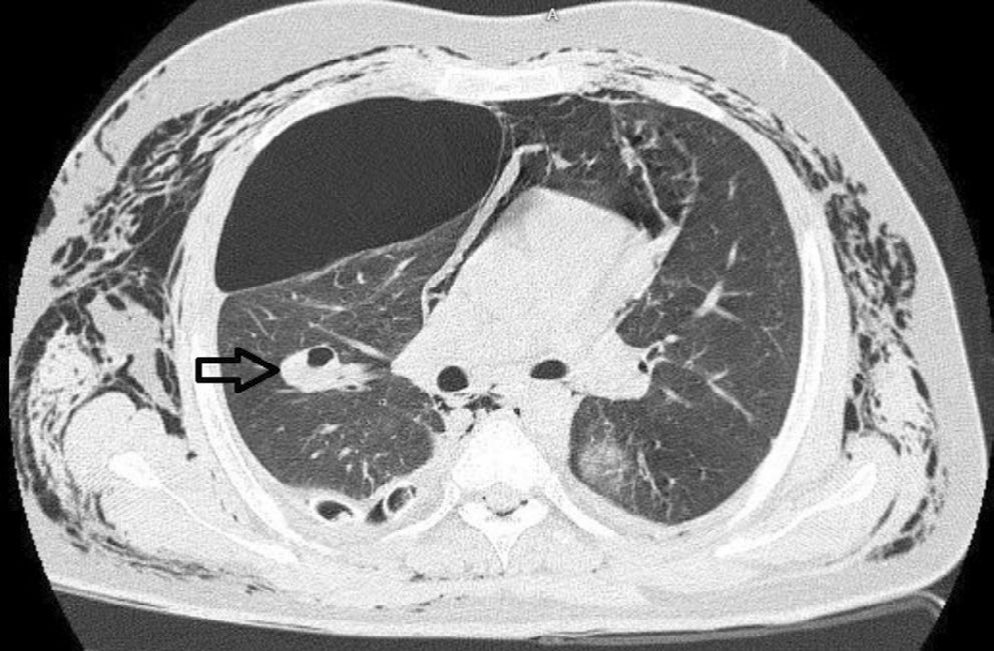
A ruptured hydatid cyst in lung CT scan (hollow arrow).

Chest computed tomography (CT) confirmed pneumothorax and pneumomediastinum and free air in the extra mediastinum (Figures 1, 2).

Pneumothorax can occur primarily or secondarily like rupture of a pulmonary cyst or bulla. Bullous lung disease is an important differential diagnosis. Pulmonary Langerhans cell histiocytosis should be considered in cystic lung disease. A ruptured bronchogenic cyst may cause pneumomediastinum. Tracheobronchial injury or esophageal rupture can lead to pneumomediastinum. Necrotizing pneumonia may result in air leakage into the pleural space or mediastinum [3].

**FIGURE 3:**
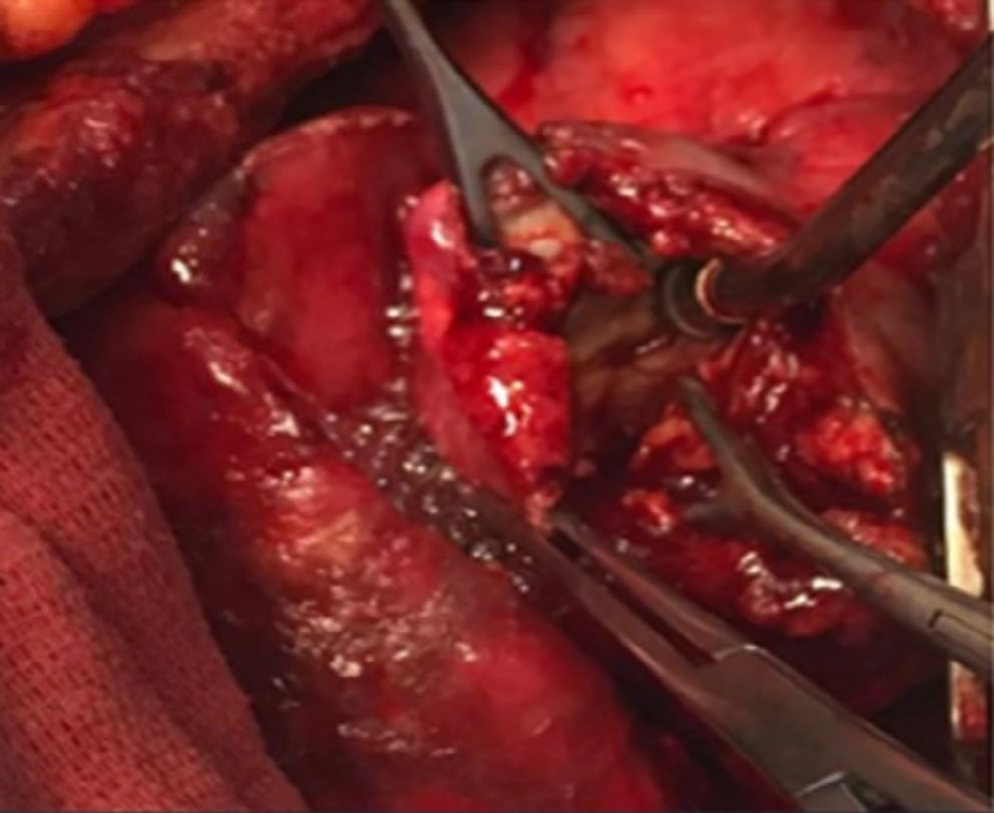
Ruptured hydatid cyst in thoracotomy.

**FIGURE 4:**
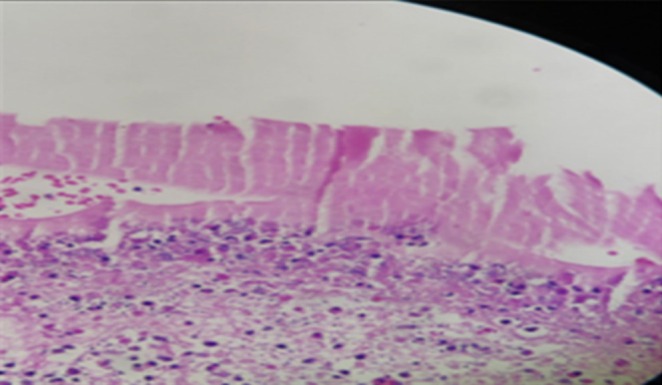
Hydatid cyst in histopathologic assessment.

The patient was admitted to the ICU, stabilized with oxygen therapy, and underwent chest tube insertion. Subsequently, after consultation with the thoracic surgeon, the patient underwent a standard right posterolateral thoracotomy for surgical management. The cystic lesion was carefully evacuated from the right upper lobe, and the resultant residual cavity was managed with capitonnage to achieve effective obliteration and support physiologic lung re‐expansion. A chest tube was inserted to ensure adequate postoperative drainage and monitoring of pleural dynamics. The procedure was completed uneventfully, with preservation of surrounding pulmonary parenchyma and satisfactory immediate postoperative stability (Figure 3). The pathologist provided the definitive diagnosis of hydatid cyst by examining the specimens (Figure 4).

## 4 Conclusion and Results (Outcome and Follow‐Up)

Postoperatively, the patient received albendazole 15 mg/kg/day (up to 400 mg), divided twice daily, in 6‐week cycles with a 2 week drug free interval to prevent hepatotoxicity, repeated for 3 months, followed by regular follow‐up. In follow‐up visit 2 weeks after discharge, the patient's complaints including chest pain and shortness of breathing were resolved.

## 5 Discussion

Hydatid cyst disease, a chronic parasitic infection in which the lungs are the second most frequently affected organ after the liver, imposes a substantial burden on human health each year [1]. Although the overall incidence of hydatid cysts has markedly decreased in high‐income countries due to improved public‐health measures and veterinary control programs, pulmonary hydatid disease continues to be reported in several endemic and transitioning regions worldwide. Importantly, even in areas where the disease is considered rare or controlled, global travel, migration, and animal‐trade dynamics underscore its persistent relevance to clinicians and public‐health systems across all geographical settings [2]. This is of particular relevance in individuals with recognized risk factors, including a history of exposure to domestic animals such as sheep or the consumption of raw or inadequately washed vegetables [3]. Complications arising from pulmonary hydatid cysts though relatively infrequent remain clinically significant, potentially severe, and occasionally fatal. Among these, rupture of a pulmonary hydatid cyst can lead to a rare but important spectrum of thoracic complications, including pneumothorax and, less commonly, pneumomediastinum [1, 4]. Pneumomediastinum may develop when air dissects from a ruptured cyst along bronchovascular sheaths into the mediastinal space or escapes from the pleural cavity. Although these complications are uncommon even within endemic regions, their occurrence carries high clinical relevance due to the potential for sudden respiratory decompensation and life‐threatening hemodynamic instability [2, 3]. The possibility of rapid clinical deterioration following rupture demands early radiologic detection and prompt, evidence‐based clinical decision making. Critically, this need extends beyond endemic regions: in a globally interconnected world, healthcare providers in both endemic and non‐endemic countries must remain aware of hydatid disease and its potential complications. Timely recognition is essential for preventing progression, reducing morbidity and mortality, and ensuring optimal patient outcomes [4, 5] The diagnosis relies heavily on radiological investigations. Chest CT remains the gold standard for identifying cyst rupture and associated complications [6]. Surgical management is the treatment of choice in complicated cases, aiming to remove cyst contents, prevent spillage, and manage complications such as pneumothorax and pneumomediastinum [6, 7]. Postoperative administration of albendazole is recommended in patients undergoing surgical removal of pulmonary hydatid cysts to reduce the risk of recurrence and to eradicate any residual parasitic elements. Albendazole therapy, typically administered for several weeks to months depending on cyst characteristics and surgical outcomes, has been shown to improve long‐term prognosis and minimize complications [8, 9].

## 6 Conclusion

Pneumothorax and pneumomediastinum are rare but severe complications following rupture of pulmonary hydatid cysts. Early clinical suspicion, radiological evaluation, and timely surgical intervention combined with antiparasitic therapy are essential to achieve favorable outcomes.

## Author Contributions

**Farzaneh Akbari:** conceptualization, investigation, writing – original draft, writing – review and editing. **Farid poursadegh:** conceptualization, investigation, writing – review and editing. **Marzie Noori:** conceptualization, investigation, writing – review and editing. **Amir Hossein Jafarian:** data curation, investigation, writing – review and editing. **Mahnaz Mozdorian:** conceptualization, data curation, investigation, writing – review and editing. **Fariba Rezaeetalab:** conceptualization, investigation, project administration, writing – review and editing.

## Funding

There is no external funding source for this case report.

## Ethics Statement

The study was approved by the Ethical Committee of Mashhad University of Medical Sciences. All procedures performed in this study involving human participants were in accordance with the ethical standards of the institutional and/or national research committee and with the 1964 Helsinki Declaration and its later amendments or comparable ethical standards.

## Consent

Written informed consent was obtained from the patient to publish this report in accordance with the journal's patient consent policy.

## Conflicts of Interest

The authors declare no conflicts of interest.

## Data Availability

The data supporting this study's findings are available from the corresponding author upon reasonable request.
